# S100A12 and hBD2 Correlate with the Composition of the Fecal Microflora in ELBW Infants and Expansion of *E. coli* Is Associated with NEC

**DOI:** 10.1155/2013/150372

**Published:** 2013-11-06

**Authors:** A. C. Jenke, J. Postberg, B. Mariel, K. Hensel, D. Foell, J. Däbritz, S. Wirth

**Affiliations:** ^1^Department of Neonatology, HELIOS Children's Hospital, 42283 Wuppertal, Witten/Herdecke University, Germany; ^2^Department of Pediatric Rheumatology and Immunology, University Children's Hospital, Münster, Germany; ^3^The Royal Children's Hospital, Murdoch Children's Research Institute, Gastrointestinal Research in Inflammation & Pathology, Melbourne, VIC, Australia

## Abstract

*Objective*. To describe the development of the gut microbiota in extremely low birth weight (ELBW) infants with and without necrotizing enterocolitis (NEC) between April 2008 and December 2009, fecal microflora was prospectively analyzed in fecal samples of all ELBW infants using real-time PCR assays. In addition, fecal inflammatory were measured. *Results*. Fecal microflora established early in ELBW infants and microbiota composition remained stable over the first 28 days of life except for the prevalence of *C. difficile* which decreased with decreasing antibiotic use. Infants who subsequently developed NEC had an increase of total bacterial count (9.8-fold) 24 h prior to clinical symptoms mainly due to the expansion of *E. coli* species (21.6-fold), whereas microbiota composition did not differ from healthy ELBW infants five days before onset of NEC. Importantly, S100A12 and hBD2 positively correlated with the total and *E. coli* bacterial CFU/g feces (*r* ^2^ 0.4 and 0.64, resp.). *Conclusions*. In summary, we found evidence for a disturbed homeostasis between the intestinal microbiome and host immunity in ELBW infants with NEC. Moreover, S100A12 and hBD2 correlate with the fecal microbiota thus linking the intestinal innate immune response to the bacterial colonization thus possibly providing a diagnostic tool in the future.

## 1. Introduction

Necrotizing enterocolitis (NEC) is a devastating disease affecting primarily premature infants. Although its etiology still remains unclear, the most widely accepted hypothesis is that NEC develops in the stressed, premature host suffering from an immature innate and adaptive immune system (reviewed in [1]) after a disruption in the intestinal barrier and translocation of bacterial endotoxins [2, 3]. Previous studies have shown that gut colonization in preterm infants is often delayed [4] and shows decreased diversity and abnormal microbiota, particularly in patients who develop NEC [5–8]. In addition, NEC has not been observed in germ-free animals [9] and infants with NEC frequently exhibit concomitant bacteremia and endotoxemia [10]. Although bacterial colonization in premature infants seems to be influenced by many host and environmental factors (reviewed in [8]), data on these processes is limited in extremely low birth weight (ELBW) infants who carry the highest risk for NEC with insufficient clinical data linking fecal inflammatory markers and antimicrobial peptides to fecal microbiota. This is particularly the case for S100A12, an endogenous damage-associated molecular pattern molecule released by activated or damaged cells under conditions of cell stress [11], and hBD2, an antimicrobial peptide and important factor in the innate host defense at the mucosal surface of the gastrointestinal tract [12]. We have previously analyzed these fecal biomarkers in ELBW infants demonstrating (i) that fecal calprotectin (fCP) levels depend on gestational and postnatal age and have a lower limit [13], (ii) that fecal S100A12 has an improved sensitivity and specificity for the detection of NEC when compared to fCP [14], and (iii) that high fecal and intestinal hBD2 concentrations, reflecting a strong intestinal immune response, were associated with a moderate course of NEC in ELBW infants [15].

Here, we now describe the development of the intestinal microbiota in ELBW infants with and without NEC. Importantly, in contrast to previous studies, we prospectively monitored the fecal inflammatory biomarkers fCP, S100A12, and hBD2 and correlated these with the composition of the intestinal microbiota in fecal samples of ELBW infants. By this means, we provide new insights into the development of intestinal microbiota in ELBW infants and their interaction with the intestinal innate immune system. 

## 2. Material and Methods

### 2.1. Patient Cohort and Sample Collection

The same cohort of extremely low birth weight infants (gestational age up to 27 + 0 weeks, born <1000 g) was used as described previously [15]. Gestational age (GA) was determined based on fetal ultrasound before the 14th week of gestation. Stool samples were prospectively collected on alternate days for the first four weeks of life. Immediately after collection samples were stored for a maximum of 48 h at 4°C and then stored at −80°C. In addition, epidemiological parameters and data about the hospital course were collected daily. NEC was defined as NEC ≥ stage 2a according to the modified Bell's criteria. Patients with spontaneous intestinal perforation (SIP) were excluded from the study. 

### 2.2. Calprotectin, hBD2, and S100A12 Analysis in Fecal Samples

Fecal calprotectin (Immundiagnostik, Bensheim, Germany) and hBD2 (Immundiagnostik, Bensheim, Germany) concentrations were determined by enzyme-linked immunosorbent assays (ELISA) [16, 17] and S100A12 concentrations by double-sandwich ELISA as described previously [18]. All analyses were performed in triplicate and normalized against controls provided by the manufacturers or the in-house S100A12 standards, respectively. Intra- and interassay coefficients of variation were <5% and <15%, respectively. Laboratory personnel were blinded to the origin and identity of patient samples. 

### 2.3. DNA Purification from Feces and Microbial Analysis with Real-Time PCR Assays

Fecal samples were 10-fold diluted in peptone/water. Bacterial DNA was isolated using the QIAamp DNA stool mini kit (Qiagen, Hilden, Germany) according to the instructions provided by the manufacturer. DNA was eluted in a final volume of 200 *µ*L. DNA from all fecal samples was subjected to real-time PCR assays for *Bifidobacteria*, *E. coli*, *C. difficile*, *Bacteroides fragilis* group, *Lactobacilli, *and total bacteria based on 16S rDNA gene sequences as described previously [19]. In all cases, the SYBR Green I method (Bio-Rad Laboratories, Hercules, CA, USA) was used:

 Bifido_fw 5′-gcgtgcttaacacatgcaagtc-3′
 Bifido_rv 5′-cacccgtttccaggagctatt-3′
 E_coli_fw 5′-catgccgcgtgtatgaagaa-3′
 E_coli_rv 5′-cgggtaacgtcaatgagcaaa-3′
 C_difficile_fw 5′-ttgagcgatttacttcggtaaaga-3′
 C_difficile_rv 5′-tgtactggctcacctttgatattca-3′
 B_fragilis_fw 5′-cggaggatccgagcgtta-3′
 B_fragilis_rv 5′-ccgcaaactttcacaactgactta-3′
 Bifidobact_inf_fw 5′-ccccgtgttgccagcggatt-3′
 Lactobac spp for 5′-agcagtagggaatcttcca-3′
 Lactobac spp rev 5′-caccgctacacatggag-3′
 Total count for 5′-tcctacgggaggcagcagt-3′
 Total count rev 5′-ggactaccagggtatctaatcctgtt-3′

PCR conditions were as follows: 95°C for 15 min, 40 cycles of (95°C for 15 seconds and 60°C for 30 seconds). Melting of PCR product was performed using a temperature gradient from 55°C to 95°C, rising in 0.5°C increments.

### 2.4. Statistical Analysis

Median quantification levels of all analyzed microbes were obtained from triplicate real-time PCR measurements. Data are presented as the median +/− interquartile range (IQR), minimum, and maximum. Testing for significant differences between groups was performed using the one-way ANOVA test for values with Gaussian distribution and the Mann-Whitney test for values without Gaussian distribution. The Kolmogorov-Smirnov test was utilized to rule out non-Gaussian distribution. Values for *P* < 0.05 were considered statistically significant. All analyses were performed using GraphPad version 5.01 (La Jolla, CA USA).

## 3. Results

### 3.1. Epidemiological Characteristics

68 ELBW infants were included in this study. Among those, 12 subsequently developed NEC and 56 had no gastrointestinal disorders (Table 1). We observed no significant differences between the disease and control group except for that infants who subsequently developed NEC tended to have a higher prevalence for antibiotic therapy at day 7 of life (Table 1). Six patients with NEC required surgery, whilst none in the other group. Mortality rate of NEC was 33% (*n* = 4), whereas in the other group no patients died. A total of 248 fecal samples were analyzed: 59 at the postnatal age of 7 days, 64 at 14 days, 59 at 21 days, and 50 at 28 days (mean 3.4 per individual, range 2 to 4) and 16 additional samples in patients with NEC in association with the disease.

### 3.2. The Intestinal Microflora in ELBW Infants Is Established during the First Week of Life, While the Use of Antibiotics Influences *C. difficile* Prevalence

**Figure 1:**
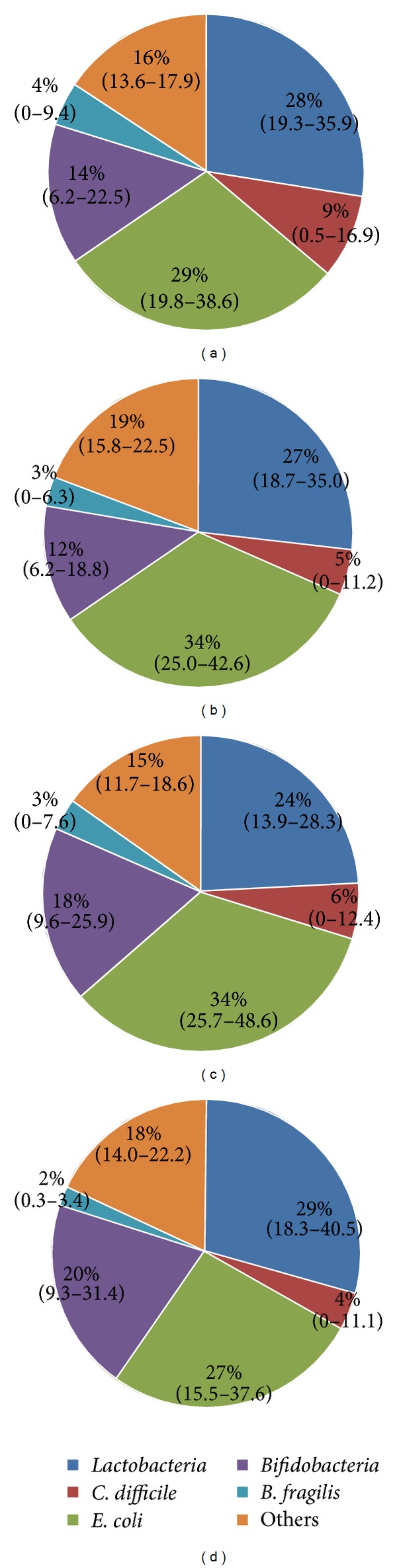
Development of the composition of the fecal microbiota in healthy ELBW infants. Proportions of the investigated microbiota at the age of one (a), two (b), three (c), and four (d) weeks. *C. difficile* prevalence declined during this period from 33 to 19% which is reflected by a similar decline of medium *C. difficile* proportion from 9 to 4%. Data is expressed as median with interquartile range.

At the age of 7 days, there were no significant differences in the median total CFU/g feces between infants who subsequently developed NEC and healthy ELBW infants except for a higher proportion of *Lactobacilli *and a lower proportion of *E. coli* in the NEC group. Also, the prevalence of *C. difficile* was slightly higher in the NEC group (Table 1). *Bifidobacteria*, *E. coli*, *Bacteroides fragilis* group, and *Lactobacilli* were detectable in all patients and in more than 98% of the samples. When further investigating factors associated with the prevalence of *C. difficile, *we did not find any influence of a specific hospital or mode of delivery, but antibiotic therapy for more than 48 h during the first week of life was associated with a higher prevalence of *C. difficile *at the age of 7 postnatal days (23 versus 35%, OR 1.78 (0.94–3.38), *P* < 0.05). At this age, the prevalence of antibiotic therapy in healthy ELBW infants was 66% and 83% in infants who subsequently developed NEC. In healthy ELBW infants, the prevalence of antibiotic therapy slowly declined thereafter to 53% at day 14 and 31% at days 21 and 28 which was mirrored by a similar decline in *C. difficile* prevalence from 33% to 25% and 19% at days 7, 14, and 28, respectively. Otherwise the overall relative composition of the fecal microflora in the reference population remained stable during the first four weeks of life (Figure 1). 

### 3.3. The Composition of the Fecal Microbiota Is Altered in Patients with NEC Shortly before Onset of Symptoms

**Figure 2:**
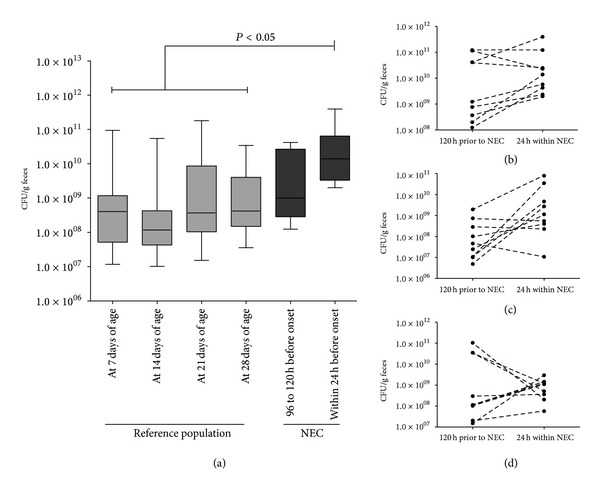
Fecal microbiota in ELBW infants with necrotizing enterocolitis. (a) Evolution of total fecal bacterial CFU/g feces in ELBW infants who subsequently developed NEC compared to the reference population. (b)–(d) Individual changes regarding total bacterial CFU (b), *E. coli* CFU (c), and *Lactobacilli* CFU (d) per g feces. Data is expressed as median with interquartile range, minimum, and maximum. A *P* value < 0.05 was considered statistically significant.

Next, we analyzed the dynamics of the composition of the fecal microbiota in infants who subsequently developed NEC. Here, we noted a marked 9.8-fold increase in the median total bacterial count shortly before onset of clinical symptoms when compared to a previous time point both in the same patients and the reference population (Figure 2(a)). This expansion was paralleled by a substantial change in the composition of the fecal microbiota mainly characterized by a steep increase in the median *E. coli* CFU/g by 21.6-fold (Table 2) and a trend for a lower median percentage of other bacteria not detected by our RT-PCR assays shortly before NEC (11.9% (9.1–19.9, range 2.2 to 24.1)) when compared to a period 90 to 120 h before onset of clinical symptoms (16.7% (12.3–24.2, range 9.6 to 37.9); *P* = 0.12). However, on an individual level, two of the twelve patients (16.7%) also showed a substantial decrease in total bacterial and *E. coli* CFU/g feces before NEC (Figures 2(b)–2(d)).

### 3.4. S100A12 and hBD2 Directly Correlate with Total and *E. coli *CFU/g Feces

**Figure 3:**
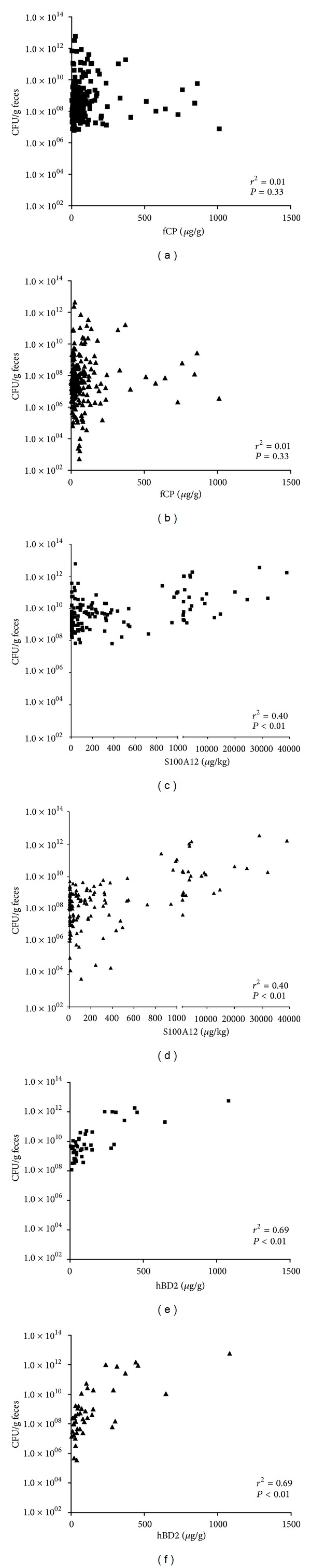
Pearson correlation analysis of S100A12, fecal calprotectin, and hBD2 with fecal microbiota. (a)-(b) Correlation between fCP and total bacterial CFU/g feces (a) and *E. coli* CFU/g feces (b). (c)-(d) Correlation between S100A12 and total bacterial CFU/g feces (c) and *E. coli* CFU/g feces (d). (e)-(f) Correlation between hBD2 and total bacterial CFU/g feces (e) and *E. coli* CFU/g feces (f). 221, 156, and 57 fecal samples were analyzed for fCP, S100A12, and hBD2, respectively.

In the last part of this study, we correlated fecal antimicrobial peptides and inflammatory markers with fecal microbiota. Here, we did not find any correlation between fCP levels and total bacterial count or any of the analyzed bacteria (Figures 3(a) and 3(b)). In contrast even though S100A12 did not correlate with CFU/g feces of Lactobacilli, Bifidobacteria or B. fragilis group, it directly correlated with the total bacterial and *E. coli* count with a correlation coefficient *r* ^2^ of 0.4 in both cases (Figures 3(c) and 3(d)). Interestingly, the antimicrobial peptide hBD2 showed a similar direct correlation with total bacterial and *E. coli* count with *r* ^2^ of 0.69 and 0.64, respectively (Figures 3(e) and 3(f)). A combination of hBD2 and S100A12 did not improve the correlation coefficient or predictive value compared to the use as single parameters.

## 4. Discussion

It is clear that intestinal microbiota have a role in the pathogenesis of necrotizing enterocolitis (NEC) and it is undoubted that a better understanding of the physiology of bacterial colonization in premature infants under *per se* nonphysiological conditions of a neonatal intensive care unit is an important tessera to elucidate mechanisms predisposing these infants to NEC. Since several previous studies revealed contradicting results, more data is needed to better characterize the exact role of intestinal microbiota in NEC pathogenesis. 

In this study, we investigate the development of the intestinal microbiota in a large cohort of ELBW infants including 12 cases of NEC. Even though we did not sequence 16S bacterial rRNA subunits but used real-time PCR assays instead, we included all major groups of intestinal bacteria like *Actinobacteria* (*Bifidobacteria*), *Firmicutes* (*Lactobacilli *and *C. difficile*), *Proteobacteria* (*E. coli*), and *Bacteroides* (*Bacteroides fragilis* group) [19]. Moreover, this is the first study to our knowledge that in parallel measured the fecal inflammatory markers calprotectin (fCP) and S100A12 and the antimicrobial peptide hBD2 and correlated these with fecal microbiota. 

Interestingly, in contrast to several previous studies [20, 21], we found *Lactobacilli, Bifidobacteria*, *E. coli,* and *B. fragilis* group in all patients after the first week of life. Particularly surprising was this observation for *B. fragilis* group since the rate of cesarean section was above 90% in our study group and colonization with *B. fragilis* group has been previously reported to depend on the mode of delivery with much lower prevalence in infants born through cesarean section [22]. Since patients in our study were included at several separated NICUs, this cannot be biased by a site-associated effect. To date, we therefore can only speculate that differences in analytical techniques and in study populations may account for this observation. The prevalence of *C. difficile* was within limits of previous reports [19–21]. We also found a direct correlation between the prevalence of antibiotic therapy and *C. difficile* colonization as described previously [19]. Regarding the further development of the intestinal microbiome, we found only minor variations during the following weeks in spite of increasing amounts of enteral and decreasing of parenteral feeding. The only exception was a decrease in the prevalence of *C. difficile* paralleled by a reduced frequency of antibiotic therapy. It therefore seems that the primary colonization has a long impact on the intestinal microbiota which is in line with previous studies on mouse models [23]. When comparing the early fecal microbiota of premature infants without gastrointestinal disorders with infants who subsequently developed NEC, we did not find significant differences but observed a tendency to higher medium CFU/g feces of *C. difficile*. However, regarding the development of NEC, we noted a rapid substantial increase in the median total bacterial CFU/g feces which mainly consisted of an increase in the median number of *E. coli*. This is in line with a previous report suggesting that premature infants might develop NEC possibly due to the insufficiency to mount an adequate adaptive immunological response to a subsequent expansion of *Proteobacteria* later in life [7]. This idea is also supported by recent data from a mouse model showing that gram-negative bacteria are associated with early stages of NEC when inflammatory markers are still negative [24]. Similarly, a high susceptibility of the premature porcine gut to *E. coli* infection has been demonstrated after delivery through cesarean section and in the absence of breastfeeding [25]. 

Furthermore, we found that the antimicrobial peptide hBD2 directly correlated with the total bacterial and *E. coli* CFU/g feces. Since hBD2 is at least partly regulated by TLR4 and MD2 signaling [26, 27], which is mainly stimulated by lipopolysaccharide (LPS), the glycolipid component of the outer membrane of gram-negative bacteria such as *E. coli, *this finding links the microbiota to the intestinal innate immunity. Importantly, we have reported earlier that ELBW infants with a particularly severe course of NEC lack an increase in hBD2 [15]. This adds further evidence to the hypothesis that an inadequate intestinal innate immune response to the intestinal microbiota plays an important role in the development of NEC. Nevertheless, we would like to emphasize that there were also patients without an increase and even a decrease in total bacterial and *E. coli* CFU/g feces prior to NEC and there was a considerable individual variation in bacterial composition as described previously [28]. Therefore, we believe that the observed changes in fecal microbiota do not represent a *sine qua non* condition for the development of NEC and are more likely just a symptom of a disturbed homeostasis at the intestinal endothelial barrier. In addition, it is important to keep in mind that fecal microbiota may not necessarily represent microbiota at the point of intestinal injury [29, 30]. 

Regarding the analysis of inflammatory markers, we found no correlation between fCP and fecal microbiota which is in line with a previous report [31]. Neutrophil-derived S100A12 in contrast correlated, similar to hBD2, with total bacterial and *E. coli* count possibly explaining why S100A12 is more sensitive and specific in predicting NEC than fCP [14].

In summary, this study provides additional evidence for the role of a differential intestinal colonization in NEC pathogenesis. Moreover, we show that the hBD2 and S100A12 directly correlate with the composition of the fecal microbiota linking it to the intestinal innate immunity. Further studies analyzing this complex interaction between gut microbes and the intestinal innate immune system might be a promising approach to gain new insights into the pathophysiology and etiology of NEC.

## Figures and Tables

**Table 1:** Epidemiological characteristics.

	Group with NEC stage ≥2 (*n* = 12)	Control group without any GI problems (*n* = 56)	*P* value
Gestational age	25.4 (24.2–27)	25.3 (23.3–27)	ns
Birth weight in g	747 (650–890)	751 (354–990)	ns
Umbilical artery pH at birth	7.34 (7.26–7.45)	7.32 (7.23–7.45)	ns
CrP at birth in mg/dL	1.52 (0.3–4.32)	1.67 (0.2–4.64)	ns
Oral feeding day 7 in mL/kg	92.4 (50–142)	84.9 (50–168)	ns
Parenteral fluids day 7 in mL/kg	77.6 (21–140)	78.8 (0–128)	ns
Mean onset of NEC (postnatal day)	14.8 (8–28)	—	—
Female (*N*)	50% (6)	58.9% (33)	ns
Delivery per caesarean section (*N*)	91.6% (11)	91.1% (51)	ns
Antibiotic therapy at day 7 (*N*)	83.3% (10)	66% (37)	ns
Exclusively breast fed at day 7 (*N*)	16.7% (2)	19.6% (11)	ns
MEDIAN LOG10 CFU per mg at day 7			
Total Bacterial count	8.5 (8.2–10.7)	8.7 (7.8–9.1)	ns
*Lactobacilli *	8.1 (7.2–9.0)	7.4 (6.9–8.2)	**<0.01**
*C. difficile* (if positive)	7.5 (4.8–11.0)	5.9 (5.2–9.6)	ns
*E. coli *	7.0 (6.0–7.6)	7.5 (6.6–7.9)	**<0.05**
*Bifidobacteria *	7.4 (6.1–7.7)	7.6 (5.5–7.9)	ns
*B. fragilis group *	6.0 (5.2–6.6)	6.0 (5.4–6.7)	ns
Others	7.7 (7.2–10.2)	7.9 (6.8–10.9)	ns
Prevalence of *C. difficile *at day 7 (*N*)	33.3% (4)	26.8% (15)	ns

**Table 2:** Intestinal microflora in infants with NEC.

	96 to 120 h prior to NEC (*N* = 12)	Within 24 h of onset of NEC (*N* = 11)	*P* value
MEDIAN LOG10 CFU per g at day 7			
Total bacterial count	9 (8.4–10.4)	9.9 (9.4–10.6)	**0.04**
*Lactobacilli *	8.7 (7.8–10.5)	8.9 (8.6–9.2)	0.49
*C. difficile* (if positive)	6.3 (4.4–8.3)	6.7 (4.1–9.3)	0.46
*E. coli *	7.9 (5.1–8.1)	9.5 (8.8–10.3)	**<0.001**
*Bifidobacteria *	7.5 (5.1–7.6)	8.3 (6.7–9.2)	0.11
*B. fragilis group *	5.8 (5.6–6.3)	7.0 (6.6–7.3)	0.28
Others	8.6 (7.7–9.9)	9.1 (8.4–9.5)	0.44
Prevalence of *C. difficile *(*N*)	33.3% (4)	27.2% (3)	
